# Breast cancer subtype and survival among Indigenous American women in Peru

**DOI:** 10.1371/journal.pone.0201287

**Published:** 2018-09-05

**Authors:** Lizeth I. Tamayo, Tatiana Vidaurre, Jeannie Navarro Vásquez, Sandro Casavilca, Jessica I. Aramburu Palomino, Monica Calderon, Julio E. Abugattas, Henry L. Gomez, Carlos A. Castaneda, Sikai Song, Daniel Cherry, Garth H. Rauscher, Laura Fejerman

**Affiliations:** 1 Division of Epidemiology and Biostatistics, University of Illinois at Chicago, Chicago, Illinois, United States of America; 2 Instituto Nacional de Enfermedades Neoplásicas, Lima, Perú; 3 Department of Medicine, University of California San Francisco, San Francisco, California, United States of America; 4 Department of Medicine, University of California San Diego, San Diego, California, United States of America; University of South Alabama Mitchell Cancer Institute, UNITED STATES

## Abstract

Latina women in the U.S. have relatively low breast cancer incidence compared to Non-Latina White (NLW) or African American women but are more likely to be diagnosed with the more aggressive “triple negative” breast cancer (TNBC). Latinos in the U.S. are a heterogeneous group originating from different countries with different cultural and ancestral backgrounds. Little is known about the distribution of tumor subtypes in Latin American regions. Clinical records of 303 female Peruvian patients, from the Peruvian National Cancer Institute, were analyzed. Participants were diagnosed with invasive breast cancer between 2010 and 2015 and were identified as residing in either the Selva or Sierra region. We used Fisher’s exact test for proportions and multivariable Cox Proportional Hazards Models to compare overall survival between regions. Women from the Selva region were more likely to be diagnosed with TNBC than women from the Sierra region (31% vs. 14%, p = 0.01). In the unadjusted Cox model, the hazard of mortality was 1.7 times higher in women from the Selva than the Sierra (p = 0.025); this survival difference appeared to be largely explained by differences in the prevalence of TNBC. Our results suggest that the distribution of breast cancer subtypes differs between highly Indigenous American women from two regions of Peru. Disentangling the factors that contribute to this difference will add valuable information to better target prevention and treatment efforts in Peru and improve our understanding of TNBC among all women. This study demonstrates the need for larger datasets of Latin American patients to address differences between Latino subpopulations and optimize targeted prevention and treatment.

## Introduction

Latina women in the U.S. have a relatively low breast cancer incidence compared to Non-Latina White (NLW) or African American women [1]. However, multiple studies have shown that the frequency of estrogen receptor(ER) negative (-) /progesterone receptor(PR) negative (-) breast cancer as well as ER-/PR-/human epidermal group factor receptor 2 (HER2) negative (-) tumors, a subtype of the disease with fewer treatment options and a poorer prognosis than other subtypes, is higher in Latinas compared to NLW [2–7]. Generally, research has shown that Latinas in the U.S. as well as Latin American women have a 20–40% higher risk of developing ER-/PR- and triple negative breast cancers (TNBC), when compared to NLW women [8–16]. These findings are important to understand the distribution of breast cancer subtypes among Latinas. However, Latinas are a heterogeneous group with ethnocultural, socioeconomic and genetic differences that are the result of diverse demographic and political regional histories. We use the term “Latina throughout the text to refer to women in the U.S. who self-identify as “Hispanic” or “Latina” and who either migrated to the U.S. or were born in the U.S. but have ancestors who were born in Latin America. Understanding the differences in breast cancer risk, subtype distribution, and outcome in different Latin American subpopulations is crucial for the development of targeted cancer prevention and treatment strategies in women of Latin American descent around the world.

There are very few studies that compare tumor subtype distribution in women from different regions within the same Latin American country [17], and no studies, to the best of our knowledge, that compare Latinas from different regions but who have similar continental genetic backgrounds. The present study compares tumor characteristic of Indigenous American Peruvian women from two different regions, the Sierra (Highlands) and the Selva (Amazonian region) in order to explore how the region of origin affects breast tumor characteristics and survival.

## Methods

### Study population

**Fig 1:**
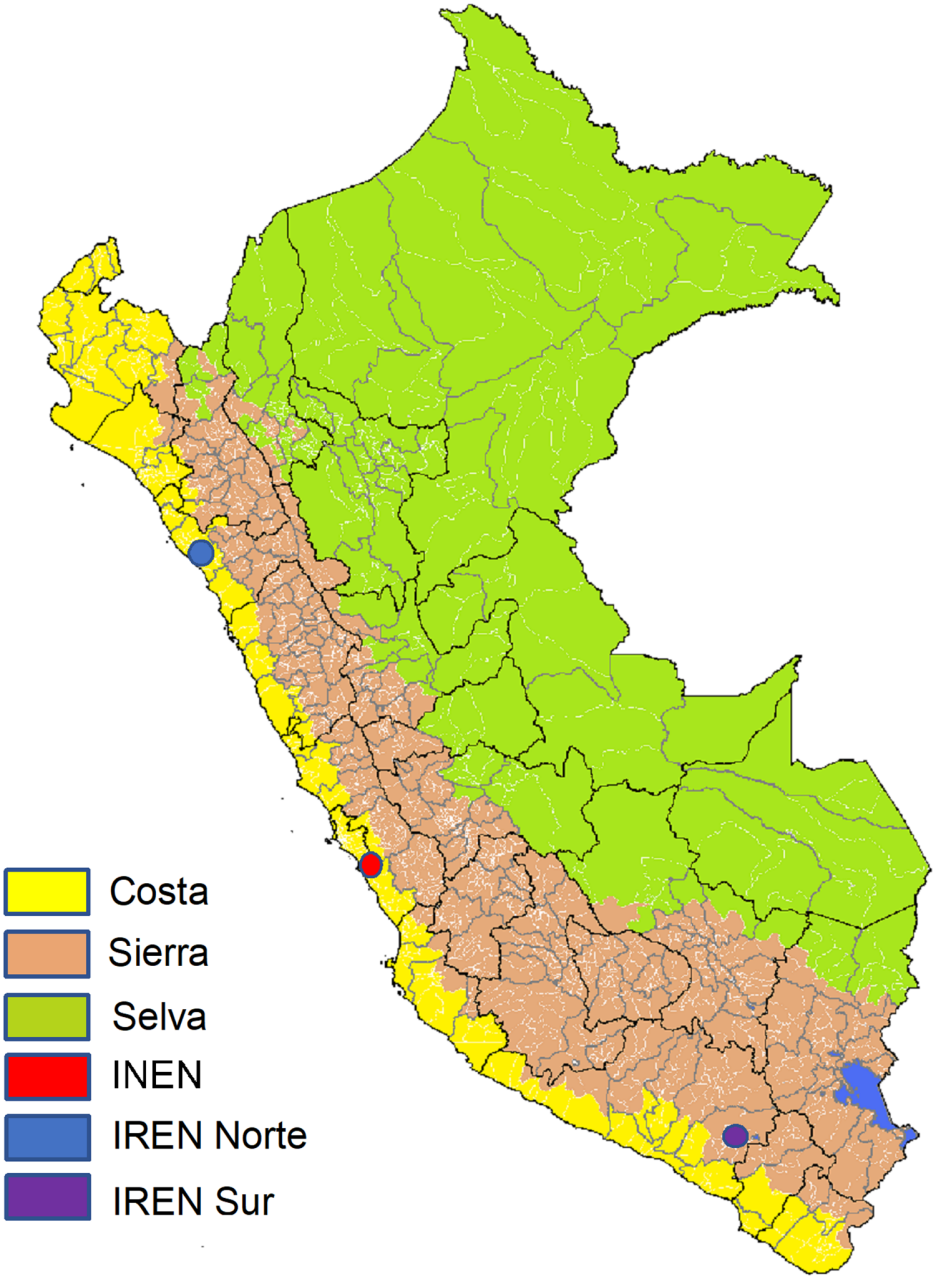
Geographical map of Peru. Different regions are colored. Green: “Selva” Amazonian region, Peach: “Sierra” Mountain region, Yellow: “Costa” Costal region, Red: “INEN location, Blue: “IREN Norte, Purple: “IREN Sur”.

We obtained data from INEN (Instituto Nacional de Enfermedades Neoplásicas) on 303 female Peruvian patients who were diagnosed with invasive breast cancer between 2010 and 2015 and who were identified as residing in areas populated by Indigenous American groups from two geographical regions: Selva and Sierra (Fig 1). Patient demographic and clinical information included age at diagnosis, birthplace, place of residence, survival status (based on periodically updated information from the Registro Nacional de Identificación y Estado Civil (RENIEC)), stage, subtype, laterality, surgery, chemotherapy, and radiation treatment, all of which were abstracted from an already existing database at INEN and anonymized before analysis.

### Breast cancer subtype definition

Breast cancers were classified into four distinct subtype categories: luminal A (ER/PR+/HER2-), luminal B (ER+/HER2+), HER2 overexpressing (ER/PR- HER2+) and triple negative (ER/PR- HER2-) based on immunohistochemistry [18].

### Statistical analysis

The statistical significance of the differences in patient and tumor characteristics between women from the Selva and Sierra regions were tested using Fisher’s exact test. Adjusted prevalence of TNBC by region was obtained using a model based standardization procedure [19,20]. Prevalence’s were adjusted by age at diagnosis (categorized as <40, 41–50, 51–65, 65+), tumor stage (categorized as stage 1, which included 7 patients who had in situ disease, stage 2 and stage 3 /4), as well as a dichotomized tumor stage variable (categorized as 0/1/2 and 3/ 4).

Kaplan-Meier survival curves were calculated for the two regions: Sierra vs. Selva (SAS 9.4). The surviving participants were censored on the date of linkage to the Peruvian mortality registry (May 2017). Survival time was measured as year of death or last linkage to mortality registry, minus year of diagnosis. We used a Cox proportional hazards model to compare overall survival between women from the Selva and Sierra regions while controlling for other clinical characteristics.

## Results

Women from the Selva region were more likely than their Sierra counterparts to have characteristics indicative of more aggressive tumors, including earlier age at diagnosis and TNBC (Table 1). The mean age at diagnosis for women from the Selva region was nearly five years earlier than for women from the Sierra region (50.1 vs. 55.0 years old at diagnosis, p = 0.001). Among the women from the Selva region, triple negative (28%) and luminal A (34%) were the most common subtypes while in the Sierra region the most common subtypes were Luminal A (46%), followed by HER2 overexpressing (18%) and TNBC (15%). Prior to adjustment for patient characteristics, women from the Selva region were also more likely to be diagnosed with TNBC (31% vs. 17%, prevalence difference (PD) = 0.14, 95% CI: 0.02, 0.27, p = 0.01); after adjustment for age and stage at diagnosis the corresponding PD was 0.14 (95% CI: 0.01, 0.27, p = 0.02) (results not tabulated).

**Table 1:** Patient and tumor characteristics among woman from the “Sierra” and “Selva” regions of Peru, 2010–2015.

Characteristics	Selva N = 71 (%)	Sierra N = 232 (%)	P-Value*
**Age, yrs**			
≤ 40	13 (18)	16 (7)	0.0089
41–50	28 (39)	75 (32)	
51–65	23 (32)	103 (44)	
>65	7 (10)	38 (16)	
Missing	0 (0)	0 (0)	
**Survival Status**			
Alive	45 (63)	183 (79)	0.0091
Dead	25 (35)	49 (21)	
Missing	1(1)	0 (0)	
**Stage**			
Stage 0/1	5 (7)	27 (12)	0.1335
Stage 2	23 (32)	93 (40)	
Stage 3/4	42 (60)	105 (45)	
Missing	1 (1)	7 (3)	
**Stage Dichotomized**			
Stage 0/1/2	28 (40)	120 (52)	0.0518
Stage 3/4	42 (59)	105 (45)	
Missing	1 (1)	7 (3)	
**Subtype**			
Luminal A	24 (34)	106 (46)	0.0293
Luminal B	10 (14)	21 (9)	
HER2	10 (14)	42 (18)	
TNBC	20 (28)	34 (15)	
Missing	7 (10)	29 (13)	
**Surgery**			
Yes	46 (65)	185 (80)	0.0155
No	24 (34)	47 (20)	
Missing	1(1)	0 (0)	
**Chemotherapy**			
Yes	59 (83)	184 (79)	0.4841
No	12 (17)	48 (21)	
Missing	0 (0)	0 (0)	
**Radiation**			
Yes	43 (61)	144 (62)	0.8196
No	28(39)	88 (38)	
Missing	0 (0)	0 (0)	

*Fisher’s Exact Test

TNBC: Triple Negative Breast Cancer

### Association between geographical region and survival

**Fig 2:**
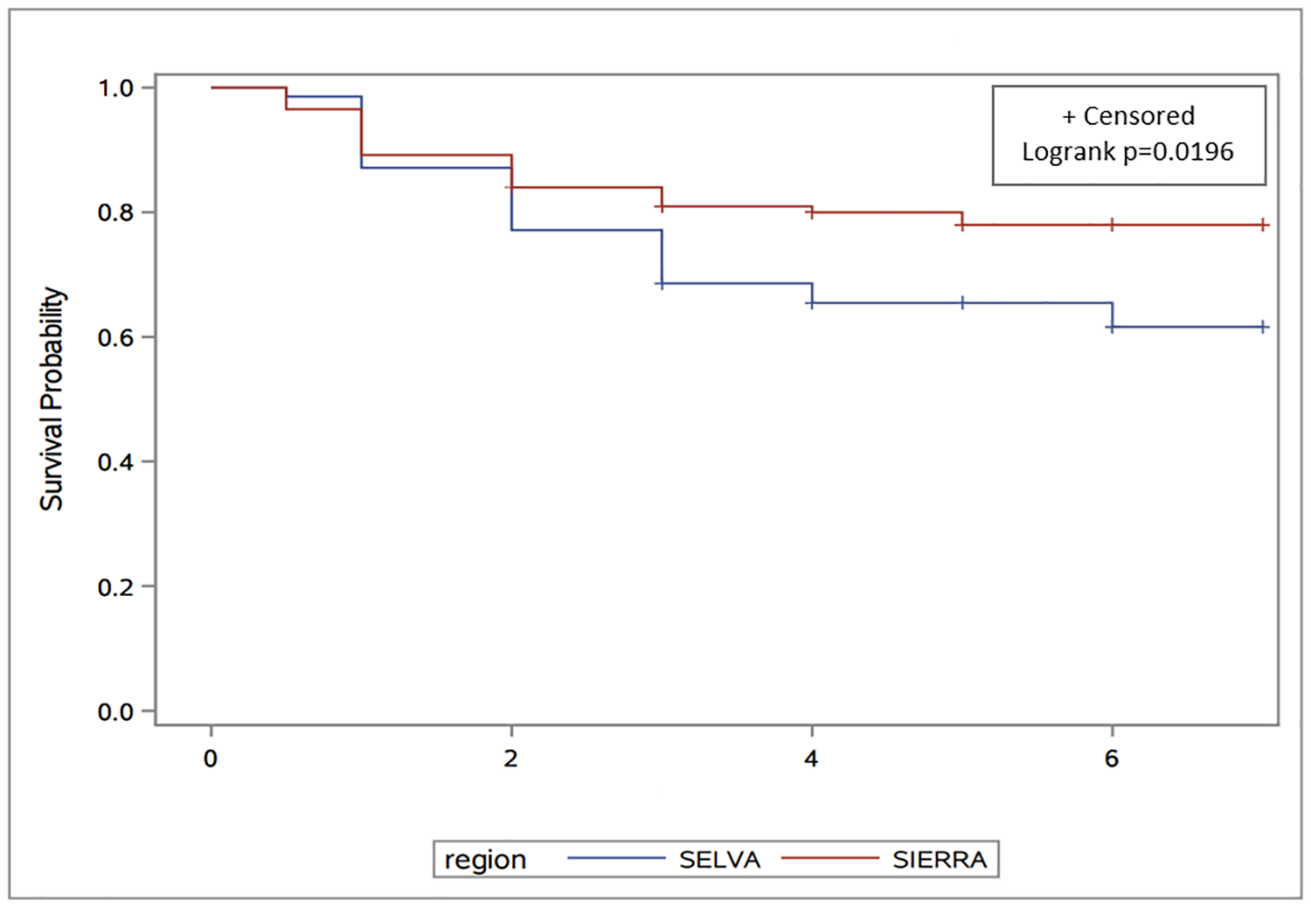
Kaplan-Meier survival curves for breast cancer patients from the Instituto Nacional de Enfermedades Neoplásicas (INEN). Comparison between women from the Selva and Sierra regions of Peru. The blue line is for the Selva region and the red line for the Sierra region.

Women from the Selva region had shorter survival than women from the Sierra (Fig 2). In the unadjusted Cox Proportional Hazards model, the hazard of mortality was 1.7 times higher in women from the Selva than the Sierra (p = 0.0253). Results from the multivariable model that included age at diagnosis, stage, tumor subtype (TNBC vs. other subtypes), and surgery, showed that region was no longer associated with survival (Table 2). The difference in the incidence of TNBC between the two regions explained most of the association between region and survival.

**Table 2:** Association between geographical region and survival in Peruvian Indigenous American Women with breast cancer, 2010–2015.

	Hazard Ratio (95 CI)	P-Value
**Univariate Analysis**		
Region (Ref. Sierra)	1.7 (1.1, 2.8)	0.0253
**Multivariate Analysis 1**		
Region (Ref. Sierra)	1.5 (0.9, 2.5)	0.1479
TNBC (Ref. No TNBC)	2.5 (1.5, 4.2)	0.0005
**Multivariate Analysis 2**		
Region (Ref. Sierra)	1.4 (0.8, 2.4)	0.1930
TNBC (Ref. No TNBC)	2.6 (1.5, 4.3)	0.0003
Age (Ref. <40 years)		0.1184
41–50	0.4 (0.2, 0.9)	
51–65	0.6 (0.3, 1.1)	
>65	0.4 (0.2, 1.06)	
**Multivariate Analysis 3**		
Region (Ref. Sierra)	1.3 (0.7, 2.1)	0.3906
TNBC (Ref. No TNBC)	2.3 (1.4, 3.8)	0.0018
Age (Ref. <40 years)		0.2234
41–50	0.5 (0.2, 0.9)	
51–65	0.6 (0.3, 1.1)	
>65	0.6 (0.2, 1.4)	
Stage Dichotomized (ref. Stage 0/1/2)	5.4 (2.8, 10.4)	<0.0001
3/4		
**Multivariate Analysis 4**		
Region (Ref. Sierra)	1.1 (0.7, 1.9)	0.6971
TNBC (Ref. No TNBC)	2.4 (1.4, 4.0)	0.0012
Age (Ref. <40 years)		0.3961
41–50	0.5 (0.2, 1.1)	
51–65	0.7 (0.3, 1.4)	
>65	0.6 (0.2, 1.5)	
Stage Dichotomized (ref. Stage 0/1/2)		0.0012
3/4	3.4 (1.7, 6.8)	
Surgery (Ref. Yes)	4.2 (2.5, 7.1)	<0.0001

## Discussion

The “Latina” identifier encompasses a heterogeneous group of individuals who differ in cultural, environmental, and ancestral genetic composition [21]. These factors contribute to differences in breast cancer risk, breast cancer characteristics and disease outcome [21]. The present analysis showed that the incidence of TNBC differs between Indigenous American women who live in the Selva (28%) and Sierra regions of Peru (15%) and that women from the Selva region have a lower probability of survival. The differences in the incidence of TNBC remained statistically significant after inclusion of tumor stage, age at diagnosis and surgical intervention status in the model. A previous analysis of INEN’s breast cancer patient population reported that the proportion of TNBC was ~20% [22], which is similar to the frequency of TNBC in our sample when taken as a whole (21%). A study that analyzed the tumor characteristics of 301 patients from the National Cancer Institute in Colombia showed a similar difference in the distribution of TNBC among Colombian regions [23]. In this study they reported that among women from the Coastal region of Colombia, TNBC represented 32% of the disease, while in the Andean region it represented 17% [17].

Differences in access to care and breast cancer awareness between women in the two regions could explain the observed disparities in stage of diagnosis, tumor subtype distribution and overall survival. INEN as well as the two regional cancer centers in Peru, are located on the Peruvian Coastal region, presenting similar logistical challenges to patients who reside in remote areas such as Indigenous American towns in the Selva or Sierra (Fig 1). The Sierra region is closer in proximity to the coast than the Selva region, therefore, women traveling from the Selva to INEN would have to travel from farther distances and potentially experience greater barriers than the women who travel from the Sierra region (average distance to INEN from the Sierra towns of Cusco, Puno, Ayacucho and Cajamarca is about 730 km while about 1830 km from the Selva towns of Tarapoto, Iquitos, Pucallpa, and Puerto Maldonado). Women who travel greater distances face more travel obstacles (i.e. travel time, fees related to travel, etc.) and are more likely to delay their presentation until the symptoms from their cancer worsen [24,25]. Additionally, the geography of Peru may create a selection bias with fewer women from the Selva region with less aggressive tumors (luminal A) travelling to INEN and dealing with the disease on their own or with the help of local healers instead. However, it is important to note that during the years of 2012–2015, Peru had a national comprehensive cancer care plan in place, called the Plan Esperanza. This plan aimed to improve access to cancer care in both remote and urban areas through the reduction of economic barriers by covering transport and accommodations of patients and an accompanying person, if necessary, and providing patients with a government subsidy to reduce out of pocket expenses [26]. The plan also sought to reduce geographical barriers through the decentralization of INEN and cultural barriers through the use of multi-causal sociocultural model for cancer control [26,27]. Therefore, even though barriers to access might explain the difference in the tumor subtype distribution between women from the two regions, a program was in place to minimize those barriers.

Variation in incidence of ER-/PR- or triple negative tumors could also be due to differences in environmental exposures or behaviors [4,28–34]. In a study by Banegas et al., residence in a low Socioeconomic Status (SES) neighborhood was significantly associated with an increased risk of diagnosis and mortality from ER-/PR-/HER2+ and TNBC [4]. This underscores the potential impact of SES, a social determinant of health, on risk factors that may be etiologically important in increasing the risk of developing ER-/PR- or TNBC. Women of low SES status or those residing in low SES neighborhoods may consume fewer healthy foods, have fewer opportunities to engage in physical activity, and have higher levels of obesity [28,31,35,36]. Additionally, low SES may also be related to younger primiparity and lack of breastfeeding, both of which have been associated with an elevated risk of TNBC [32–34]. These findings are relevant for U.S. Latinas, but might not apply to women who reside in Latin America. Therefore, studies addressing disparities in different Latin American countries should take into account the particular sociocultural and socioeconomic structures and factors that are associated with them [37–39].

Lastly, variation in tumor subtype distribution could be partly due to differing frequencies of predisposing genetic alleles between populations, as exposure to known risk factors may explain some [8,40–42] but not all [13,43,44] of the differences observed. There are very few studies that compare tumor subtype distribution in women from different regions within the same Latin American country [23], and no studies, to the best of our knowledge, that compare Latinas from different regions but who have similar continental genetic background. Future research should focus on the discovery of subcontinental genetic differences (e.g. genetic differences between Aymaras, Quechuas, Awajun, Shipibo-Conibo, etc.) that might, in part, explain the distribution of breast cancer tumor subtypes and survival in diverse Latin American populations. Although this study adds valuable information regarding breast cancer subtypes among Latina women, limitations were present. Tumor subtype information was missing for 12% of the total eligible participants which resulted in 13% and 10% missing from the Sierra and Selva regions, respectively. In addition, breast cancer-specific survival analysis was not implemented as we did not have access to information on cause of death. We also lacked detailed dates of diagnosis and mortality, and relied on year of diagnosis and year of death to construct survival times. Misclassification of survival time is likely to have been non-differential with respect to region and therefore the increased mortality hazard for Selva vs. Sierra is unlikely to be due to misclassification. The higher prevalence of TNBC among women from the Selva region appeared to be the main driver of the survival difference by region that we observed. Finally, women were identified as Indigenous American based on their reported residence, since genetic ancestry estimations were not available. Given that the average proportion of Indigenous American ancestry in Peru is about 70–80% [45]), it is reasonable to assume that women who reside in well known “pueblos indígenas” (Indigenous populations) are likely to have a high average proportion of Indigenous American ancestry.

## Conclusion

Our results suggest that the distribution of breast cancer subtypes differs between highly Indigenous American women from the Selva and Sierra regions of Peru and that this difference partly explains the observed disparity in the risk of mortality. Women from the Selva region are more likely to be diagnosed with TNBC and less likely to survive compared to women from the Sierra region. Understanding the factors that contribute to the observed disparities will not only add valuable information to better target prevention and treatment efforts in Peru, but will also add to our overall understanding of what factors contribute to the development of TNBC not only among women of Latin American origin, but among all women.

## Supporting information

S1 Dataset Supporting data. (XLSX)
